# Cinchonain Ia Shows Promising Antitumor Effects in Combination with L-Asparaginase-Loaded Nanoliposomes

**DOI:** 10.3390/pharmaceutics15051537

**Published:** 2023-05-19

**Authors:** Thi Nga Nguyen, Thi Phuong Do, Thi Cuc Nguyen, Ha Phuong Trieu, Thi Giang An Nguyen, Thi Thao Do

**Affiliations:** 1 Institute of Biotechnology, Vietnam Academy of Science and Technology, 18 Hoang Quoc Viet Road, Cau Giay, Hanoi 100000, Vietnam; 2 Faculty of Biology, College of Education, Vinh University, 182 Le Duan St., Vinh City 460000, Vietnam; 3 Vietnam Academy of Science and Technology, Graduate University of Science and Technology, 18 Hoang Quoc Viet Road, Hanoi 100000, Vietnam

**Keywords:** CALs, cancer stem cell, cinchonain Ia, L-asparaginase, nanoliposome, NTERA-2 cells, tumorsphere

## Abstract

Cancer is among the leading causes of death worldwide, with no effective and safe treatment to date. This study is the first to co-conjugate the natural compound cinchonain Ia, which has promising anti-inflammatory activity, and L-asparaginase (ASNase), which has anticancer potential, to manufacture nanoliposomal particles (CALs). The CAL nanoliposomal complex had a mean size of approximately 118.7 nm, a zeta potential of −47.00 mV, and a polydispersity index (PDI) of 0.120. ASNase and cinchonain Ia were encapsulated into liposomes with approximately 93.75% and 98.53% efficiency, respectively. The CAL complex presented strong synergistic anticancer potency, with a combination index (CI) < 0.32 in two-dimensional culture and 0.44 in a three-dimensional model, as tested on NTERA-2 cancer stem cells. Importantly, the CAL nanoparticles demonstrated outstanding antiproliferative efficiency on cell growth in NTERA-2 cell spheroids, with greater than 30- and 2.5-fold increases in cytotoxic activity compared to either cinchonain Ia or ASNase liposomes, respectively. CALs also presented extremely enhanced antitumor effects, reaching approximately 62.49% tumor growth inhibition. Tumorized mice under CALs treatment showed a survival rate of 100%, compared to 31.2% in the untreated control group (*p* < 0.01), after 28 days of the experiment. Thus, CALs may represent an effective material for anticancer drug development.

## 1. Introduction

Cancer is a disease group characterized by uncontrolled growth and the wide spread of abnormal cells in the body. According to a report by WHO, cancer was a major cause of death for 10 million patients in 2020. Furthermore, the incidence and death of cancer are predicted to rise by 28.4 million new cases in 2040 [1]. However, chemotherapy for cancer diseases has encountered many obstacles due to widespread drug resistance, unexpected side effects, and the biodegradation of drugs.

Nanoparticle formulas such as liposomes represent an effective solution for minimizing biodegradation and systematic immunological reaction occurrence. The main components of liposomes are phospholipids, which are similar to cell membranes and therefore protect encapsulated drugs from degradation, help targeted delivery, and provide control, leading to improved therapy and a reduction in required doses and adverse effects. Liposomes have been used to encapsulate various types of drugs, including hydrophilics and hydrophobics. They are also convenient, allowing the simultaneous administration of combinations of two or more drugs (multi-drugs) in a single delivery system to improve antitumor therapy effects, accumulate drugs in tumor tissues, and extend the blood circulation time [2]. Approximately 20 liposome-based medicines have recently been approved for clinical application worldwide. These drugs have shown significantly improved anti-cancer effects and reduced adverse effects [3].

Among commercial drugs used in cancer therapy, L-asparaginase (ASNase) is a homotetrameric enzyme that is widely applied to treat acute lymphoblastic leukemia (ALL) and non-Hodgkin’s lymphoma through the catalysis of L-asparagine into aspartic acid and ammonia, decreasing the availability of L-asparagine for cancer cell growth [4,5]. However, ASNase has limited clinical applications due to hypersensitive reactions, unfavorable immune responses, short circulatory half-life, and undesired side effects such as fever, chills, skin rashes, anaphylaxis, and severe allergic reactions [6,7,8]. Loading ASNase into liposomes has effectively solved most of these issues [9,10,11,12]. In addition, drug combinations are also a promising approach for both solving side effects and increasing the anticancer activity of ASNase [13]. ASNase combined with decitabine reveals cytotoxic activity in acute T leukemia (T-ALL), when detected with a specific genetic marker [14]. The combination of this enzyme with vincristine and prednisolone in intravenous injection has also shown excellent results for the treatment of leukemia. Drugs such as dexamethasone and methotrexate have also been used in combination with L-asparaginase in cancer treatment [15]. Therefore, the combination of L-asparaginase with other active ingredients or drugs to improve their therapeutic effects is a promising avenue for research.

Cinchonain Ia, which is isolated from the plant *Peltophorum pterocarpum* (DC.) K. Heyne, exhibits anti-Alzheimer and antioxidant activity [16]. It markedly inhibits Aβ aggregation, which plays an important role in Alzheimer’s disease [17]. It also exhibits anti-inflammatory activity by inhibiting interleukin-1β, an important mediator of the inflammatory response and of a variety of cellular activities including cell proliferation, differentiation, and apoptosis [18]. Therefore, in this study, we encapsulated cinchonain Ia and ASNase into nanoliposomal complex particles (CALs). Then, CALs will be evaluated for their enhanced anticancer efficacy in vitro and in vivo. 

## 2. Results

### 2.1. Effects of Drug Combination and CI Determination

The antiproliferative potency of the ASNase/cinchonain Ia combination was demonstrated in the various ratios of ASNase and cinchonain Ia. As shown in Table 1, the activity of ASNase and cinchonain Ia depended on either the cell line or ratio of drugs. The IC_50_ value of ASNase on the NTERA-2 cell line was 10.3 mIU/mL, while this value was three-fold higher on A549 cell line. Similarly, cinchonain Ia also expressed stronger activity on NTERA-2 cells when compared with that on A549 cells. The IC_50_ of cinchonain Ia on A549 cells was four-fold higher than that on NTERA-2 cells. The result claimed that cinchonain Ia and ASNase were more toxic on the NTERA-2 cell line than on A549 cells.

The amalgamation of ASNase and cinchonain Ia reduced the IC_50_ of either cinchonain Ia or ASNase itself on both A549 and NTERA-2 cells at numerous ratios of drugs. The CI values ranged from 0.71 to 0.81 on A549 at ratios of ASNase (IU):cinchonain Ia (µg) from 1:12.5 to 1:100, indicating a moderate synergistic effect of the combination on this cell line. However, the ratio of 1:200 presented an increasing CI of 0.90, which suggested an additive effect. At the same time, the ratio of 1:25 to 1:100 expressed clear synergy due to the CI of 0.57–0.76 on NTERA-2 cells. Nonetheless, moving out of this range (ratios 1:12.5 and 1:200) involved increasing the CI value to over 1.1, which intimated the antagonism effect of the combination.

### 2.2. Physicochemical Characteristics of L-Asparaginase-Loaded Cinchonain Ia Liposomes

**Figure 1:**
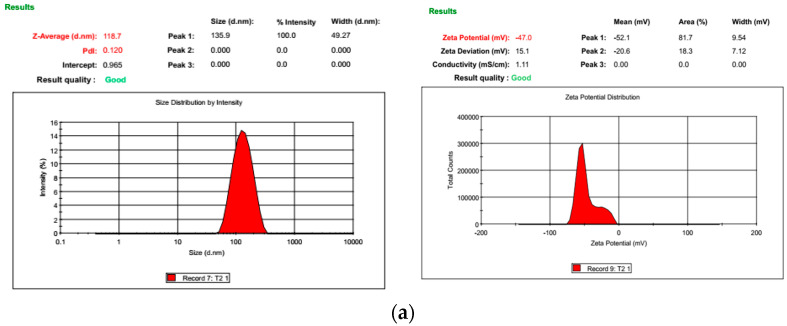

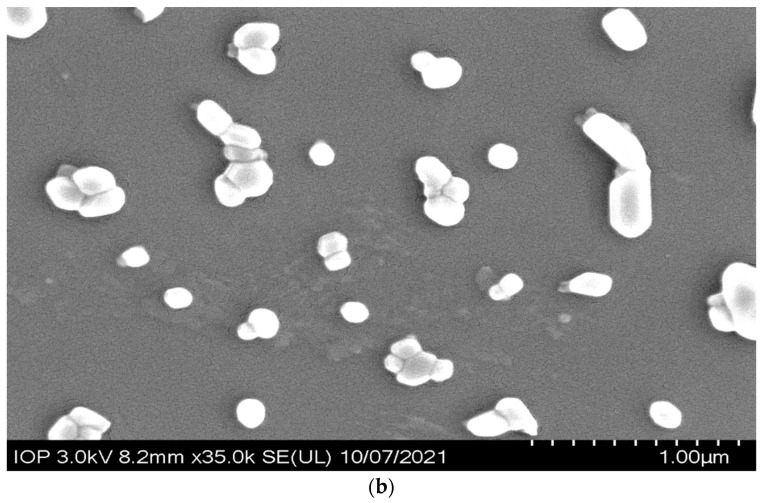
The characteristics of obtained CAL nanoliposomes: (**a**) the physical parameters of CALs using Zetasizer Nano ZS (Malvern Panalytical Ltd., Malvern, UK); (**b**) Field Emission Scanning electron microscopy (FESEM) morphological image of CALs (HITACHI S–4800 system, Hitachi Medical Corporation, Tokyo, Japan).

Lipid components including lecithin, cholesterol, and D-α-tocopherol polyethylene glycol succinate (TPGS) were used to prepare the liposomes. Cinchonain Ia and ASNase were incorporated into liposomes using the traditional Bangham method. All liposomal formations were uniform in size (<150 nm) (Figure 1a). The polydispersity index (PI) of each liposome was smaller than 0.20, with negative zeta potential values regardless of drug loading (Table 2). Both uni-liposomes and dual liposomes showed high drug-encapsulation efficiency, with 93.75% and 98.53% for ASNase and cinchonain Ia, respectively, in dual-liposome form. There was no significant difference between the uni-liposome and dual-liposome forms of either drug. The SEM morphology of CALs presented small unilamellar vesicles, including both likely rod-shape and spherical forms (Figure 1b).

### 2.3. Cellular Uptake Capacity of CALs

**Figure 2:**
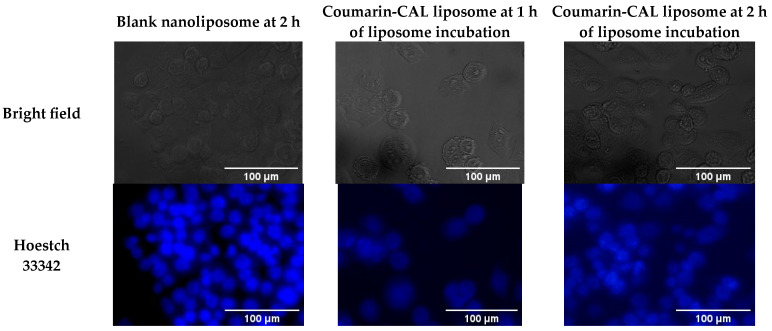

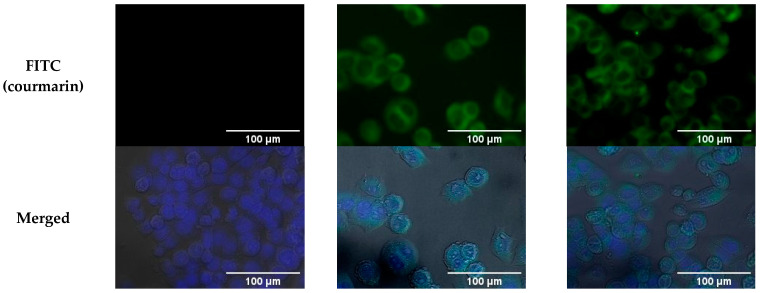
Microscopic images of NTERA-2 cells after 1 h or 2 h incubated with blank nanoliposome or coumarin-6 probing CAL nanoliposome, observed under a fluorescent microscope system—Olympus ScanR 100.

The NTERA-2 uptake capacity toward CAL nanoliposomes, which were incorporated with coumarin-6, was assessed. As shown in Figure 2, after 1 h of incubation with NTERA-2 cells, the intensity of the fluorescent appeared stronger than the control, indicating that the CAL liposome had entered into the cytoplasm. Moreover, the intensity of the fluorescent was presented even more strongly at the 2 h tested time-point, meaning that the CAL complex continuously embarked and accumulated in the cytoplasm. 

### 2.4. CAL Anti-Proliferation Activity in NTERA-2 Cells in Two-(2D) and Three-Dimensional (3D) Cultures

**Figure 3:**
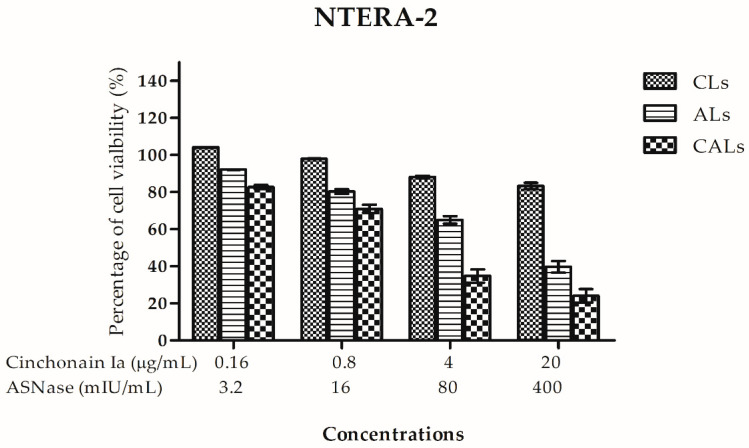
Effects of CAL dual nanoliposomes, CL uni-liposomes or AL uni-liposomes on the NTERA-2 cell viability in a monolayer cell culture model after 72 h of incubation using MTT anti-proliferative assay.

Due to NTERA-2 cells being sensitive to both ASNase and cinchonain Ia, we evaluated the potential of liposomes in this cell line in continuous 2D and 3D cultures. In the 2D cell culture, CALs exhibited strong anti-proliferative effects in the NTERA-2 cancer stem cell line, with IC_50_ = 46.0 ± 6.0 mIU and 2.30 ± 0.29 µg/mL for ASNase and cinchonain Ia, respectively. ASNase liposomes showed weaker cytotoxicity than CALs, whereas cinchonain Ia liposomes expressed less cytotoxic potency than all other liposomes (Figure 3). The IC_50_ value of CALs (IC_50_ = 22.3 ± 3.0 mIU) was more than 8.69- and 4.84-fold higher than those of either cinchonain Ia or ASNase uni-liposomes, respectively.

**Figure 4:**
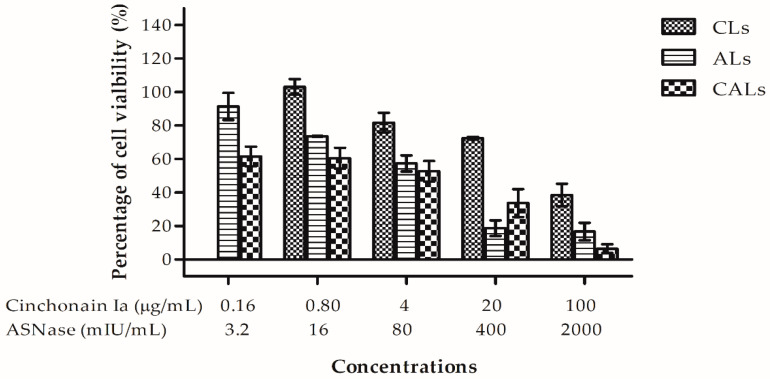
Antiproliferation activities of CAL dual nanoliposomes, CL uni-liposomes or AL uni-liposomes on the NTERA-2 cell viability in a 3D cell culture model after 72 h of incubation using MTT anti-proliferative assay.

In 3D cell culture, liposomes affected cell viability in the tumorsphere in a dose-dependent manner (Figure 4). Similar to their activity in 2D cell culture, liposome cytotoxicity on 3D spheroids occurred in the order cinchonain Ia liposomes < ASNase liposomes < CALs. CALs exhibited IC_50_ values of 1.86 ± 0.37 µg/mL for cinchonain Ia and 37.0 ± 7.0 mIU for ASNase. By contrast, cinchonain Ia and ASNase liposomes had IC_50_ values of 57.37 ± 1.34 µg/mL and 90.0 ± 9.0 mIU/mL, respectively. These results indicate the superior efficiency of dual liposomes, i.e., CALs, with more than 30- and 2.5-fold higher cytotoxic activity than cinchonain Ia and ASNase liposomes, respectively.

### 2.5. In Vivo Antitumor Activity of CALs

**Figure 5:**
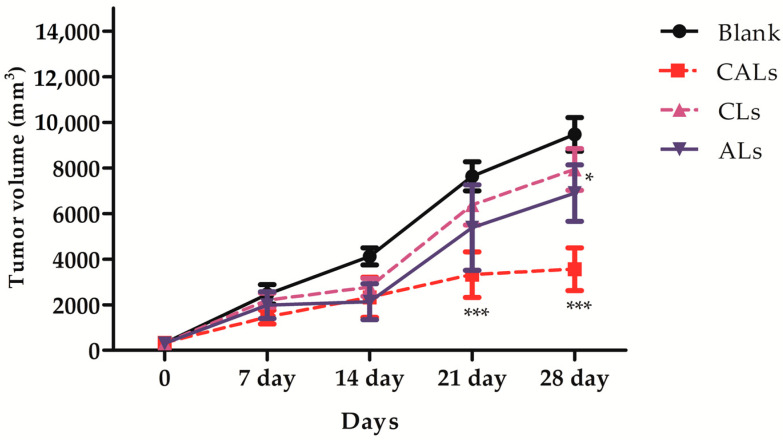
The anti-tumor capacities of CAL dual nanoliposomes, CL uni-liposomes or AL uni-liposomes on BALB/c mice harboring malignant tumor induced by LLC cells (*n* = 6). CAL liposome at doses 3 IU/mouse significantly inhibited tumor growth after 21 and 28 days compared with the negative control (blank liposome) (*** *p* < 0.001 and * *p* < 0.05, respectively). Error bars represent standard error (SE).

An in vivo study was performed to evaluate the antitumor activity of CALs in a Lewis lung carcinoma (LLC) tumor-induced mouse model. Nanoliposomes were injected at a fixed dose of 5 mg/kg body weight cinchonain Ia and/or 100 IU/kg ASNase every 2 days, for a total of seven injections. No significant difference in body weight was detected among the treatment and control groups (Table 3), demonstrating that these liposomes are not toxic to animals. Tumor volume was reduced at different time points in all treated groups during the study, depending on the liposomal formulation (Figure 5 and Table 4). Cinchonain Ia liposomes displayed only mild anti-tumor efficacy, inhibiting approximately 16.24% of the tumor growth by the end of the experiment. ASNase liposomes exhibited better anti-tumor activity than cinchonain Ia liposomes, inhibiting 27.21% of the tumor development. Notably, animals treated with CALs exhibited significant tumor growth suppression compared to both uni-nanoliposomes, inhibiting 62.49% of tumor development compared to the control group.

## 3. Discussion

Drug combinations in cancer therapy were first introduced in 1965 by Frei et al. [19]. This approach confers many benefits, including enhanced therapeutic effectiveness compared to monotherapy approaches and reduced drug resistance while maintaining or even increasing anticancer effectiveness in a synergistic or an additive manner [19,20]. For these reasons, ASNase has been used as a component in combination drug therapies to treat acute lymphoblastic leukemia and non-Hodgkin’s lymphoma [21]. Although ASNase has some side effects, it tends to enhance treatment efficiency. In this study, we amalgamated ASNase with cinchonain Ia to boost anticancer activity. The IC50 values of ASNase and cinchonain Ia were approximately two- to fourfold higher in A549 cells than in NTERA-2 cancer stem cells, whether treated singly or in combination (Table 1), indicating that these compounds had stronger effects on NTERA-2 cells than on A549 cells. The combination of these two drugs affected A549 and NTERA2 cells with CI values ranging from 0.5 to 0.8, revealing a synergistic effect of combining ASNase and cinchonain Ia. L-ASNase concentrations were markedly reduced in the combined formulation with cinchonain Ia, producing a toxicity similar to that of ASNase alone (*p* < 0.05). The synergistic activity of combined treatment was most effective at an ASNase (IU): cinchonain Ia (µg) ratio of 1:50. Therefore, we applied this proportion in the liposome preparation to amplify the anticancer effect of the combined treatment.

According to Immordino et al. (2006), a diversity of structural components, sizes, and zeta voltages allows the development of a wide range of nanoliposome designs to combine multiple drugs [22]. These flexible nanoliposome structures also increased its bioavailability by enhancing the uptake capacity of target organs and avoiding depletion by the mononuclear phagocyte system to raise the drug’s half-life. Thus, using liposomes to co-deliver multiple anticancer agents is a promising strategy to improve anticancer potential by reducing concentration demand and unexpected side effects, while increasing synergic drug expression at tumor sites [23]. Surfactants can also be used to modify the liposome surface, enhancing bioavailability. In this study, CALs were coated with TPGS, which is a water-soluble derivative of vitamin E produced by PEGylate, to obtain an amphiphilic structure comprising a hydrophilic polar head and a lipophilic alkyl tail [24]. This biocompatible macromolecule exhibits enhanced solubility, cellular permeation, circulation time, nanostructure stability, and drug release control, and has therefore been reported to offer numerous benefits including enhanced antioxidant and anticancer bioactivity in many hydrophobic agents [25,26,27]. Using this excipient as a surfactant improved the size of homogeneous cinchonain Ia and ASNase uni-liposomes and CAL dual liposomes to approximately 120 nm and increased their zeta voltage and cellular uptake capacity compared to non-TPGS-coated liposomes. This study is the first to manufacture cinchonain Ia uni-liposomes or cinchonain Ia/ASNase-loaded dual nanoliposomes. We prepared ASNase liposomes in a previous study using another coating agent, PEG200, which resulted in smaller particles [10].

Liposome cytotoxicity was evaluated in NTERA-2 cells in both 2D and 3D models. The IC50 of co-loaded liposomes was significantly reduced in both experimental models compared to uni-loaded liposomes. The CI of the multiple-drug liposome was <0.32 in the 2D model and 0.44 in the 3D model. A CI index < 0.4 indicated strong synergy [28]. Therefore, the CALs produced in this study represent strong synergistic anticancer potency. The outstanding efficiency of these multi-drugs incorporated into liposomes was demonstrated in both in vitro and in vivo experiments.

Both cinchonain Ia and ASNase uni-liposomes exhibited mild anticancer activity at the experiment dose, whereas CALs dramatically inhibited tumor growth at different time points compared with single-drug treatment in LLC-induced mice. Notably, only a half dose of ASNase (3 IU) was required to achieve significant results, compared to a previous study. As reported by Thao et al., a dose of 6 IU/mouse exhibited only an approximate 32% decrease in tumor growth by the end of the experimental period, whereas a dose of 3 IU of L-asparaginase combined with cinchonain Ia in the form of a co-loaded nanocomplex had nearly double the inhibitive effect on tumor development in terms of percentage growth [10]. At the end of the experiment, after 28 days, the number of survival mice in the CALs treated group was recorded as 100%, in comparison with 31.2% of the control group, presenting a significant protective effect for tumorized mice as well.

## 4. Materials and Methods

### 4.1. Materials

L-asparaginase was purified from the GB911 bacterial strain, which was isolated from marine sediments of the Khanh-Hoa Sea in Vietnam [29]. Cholesterol, soybean lecithin, fetal bovine serum (FBS), gentamicin, and D-alpha tocopheryl polyethylene glycol succinate (TPGS) were obtained from Sigma Chemical Co. (St. Louis, MO, USA). Dulbecco’s modified Eagle’s medium and nonessential amino acid (NAA) and L-glutamine were purchased from Invitrogen (Carlsbad, CA, USA). Cinchonain Ia with 99% purification was kindly provided by Prof. Trinh Thi Thuy (Institute of Chemistry, Vietnam Academy of Science and Technology) [30].

Male and female albino BALB/c mice (12 weeks old) were from the Institute of Biotechnology, Vietnam Academy of Science and Technology (VAST, Hanoi, Vietnam). The animals were caged in a temperature-controlled room on a 12 h light/12 h dark cycle with food and water ad libitum. Experiments were performed in accordance with Vietnamese Ethical Laws, European Communities Council Directives of 24 November 1986 (86/609/EEC) guidelines and approval from the Scientific Council of the Institute of Biotechnology, VAST, for the care and use of laboratory animals.

### 4.2. Preparation of L-Asparaginase Nanoliposome Loaded Cinconain Ia (CALs)

The liposomes were prepared according to the method by Meers et al. (2008) [31], with slight modification. Briefly, soya bean lecithin and cholesterol were mixed in methanol solution at a molar ratio of 9:1 along with 5,2 mM D-alpha tocopheryl polyethylene glycol succinate (TPGS) and 2.5 mg cinchonain Ia. The mixture was stirred and then dried using a rotary evaporator under an aspirate vacuum at 40 mmHg. The thin-film layer was flushed with nitrogen gas to remove a trace of organic solvent. The lipid layer was hydrated with a small volume (0.2 mL) of normal saline solution containing 50 IU ASNase for 5 min and then 5 mL of normal saline solution was added to form a multilamellar vesicle (MLV). Subsequently, the multilamellar vesicles (MLV) were sonicated for 10 min to reduce the size of the particles. Finally, the drug-loaded liposome was filtered through a 0.22 µm membrane filter to collect liposomes. The sharply followed protocols were applied to prepare either cinchonain Ia liposomes (CLs) or L-asparaginase liposome (ALs).

### 4.3. Encapsulation Efficiency of L-Asparaginase and Cinchonain Ia in Liposomes

The encapsulation efficiency of L-asparaginase was determined following the previous report of Thao et al. (2019) [10], while the amount of Cinchonain Ia encapsulated in the liposome was evaluated by the HPLC method. The liposome was dissolved in DMSO 100% before vortexing for 15 min. Then, 10 µL of aliquot was injected into the HPLC column. The encapsulation efficiency was calculated according to the following formula:

(1) $$EE\%=\frac{Cf}{Ci}\times 100$$

where *Cf* is the amount of encapsulated drug in liposomes measured after, and *Ci* is the amount of drug added to the lipid mixture.

### 4.4. Size Distribution and Particle Morphology Analysis

The size distribution and zeta potentials of liposomes were measured with a Zetasizer Nano-Z (Malvern Panalytical Ltd., Malvern, UK) at 25 °C. The morphology of the liposomes was observed using field emission scanning electronic microscopy (FESEM) (HITACHI S-4800 FESEM system).

### 4.5. Cell Culture

The NTERA-2, A549, and Lewis Lung Carcinoma (LLC) cancer cell line were a gift of provided by Dr. P. Wongtrakoongate, Mahidol University, Thailand, and Prof. J. Maier, Milan University, Italy. These cell lines were grown in DMEM medium supplemented with 10% fetal bovine serum (FBS), 2 mM L-glutamine, and 50 µg/mL gentamicin. They were all maintained in a 100% humidifying incubator at 37 °C and 5% CO_2_.

### 4.6. Determination of Cellular Uptake

Cellular liposome uptake capacity was evaluated by using coumarin-6 as fluorescent prober (presented signal as FITC). NTERA-2 cells were seeded on the 6-well plate at a density of 3 × 10^5^ cells/well and maintained in a CO_2_ incubator overnight. The cells were then treated with coumarin-probed liposome at a concentration of 10 µg/mL of coumarin for 2 h. After that, the supernatant was removed and cells were washed with cold PBS three times. Formaldehyhe 10% was added to each well to fix cells. After removing the fixation solution, the nuclei of cells were stained with Hoechst 33342 for a further 15 min. The morphology of cells was observed under a Olympus ScanR 100 fluorescent microscope system (Olympus Europa SE & Co.KG, Hamburg, Germany).

### 4.7. In Vitro Cytotoxicity Analysis

The antiproliferative effect of liposomes against cancer cell lines was evaluated using an MTT assay [32]. Briefly, cells were seeded in a 96-well plate at a density of 1 × 10^4^ cells/well and treated with samples for 72 h. In experiments determining the effect of samples when combining drugs, cinchonain Ia was mixed with ASNase at different ratios before adding to the cells. After the incubation time, the medium was discarded and 50 µL fresh MTT (5 mg/mL) solution was added to each well for 4 h at 37 °C. Then, the MTT solution was discarded before adding 100 µL DMSO 100% per well to dissolve the formazan crystal. The absorbance was measured at 570 nm using an automated microplate reader (ELx800, BioTek Instrument, Winooski, VT, USA). The effect of liposomes on cell viability was calculated with the following formula:

(2) $$\mathrm{Percentage}\;\mathrm{of}\;\mathrm{growth}\;\mathrm{inhibition}=100\%-\frac{\mathrm{OD}\;\left(\mathrm{sample}\right)-\mathrm{OD}\;\left(\mathrm{blank}\right)}{\mathrm{OD}\;\left(\mathrm{control}\right)-\mathrm{OD}\;\left(\mathrm{blank}\right)}\times 100\%$$

where OD (control): absorbance of well with untreated cells; OD (sample): absorbance of well with treated cells; and OD (blank): absorbance of well was medium only.

#### Evaluation of Inhibitive Efficacy of CALs in Tumorspherical Models

For 3D culture, 3000 cells per well were seeded in a 96-well, agarose-coated plate. The cells were incubated at 37 °C, 5% CO_2_ for 72 h to form tumorspheres. Then, the cells were treated with optimized doses of cinchonain Ia liposome (CLs), L-asparaginase liposome (ALs) or L-aspaginase combined cinchonain Ia liposome (CALs) and were further incubated for 7 days. The experiments were performed in triplicate. After 7 days, the medium was discarded and 50 µL of MTT solution (1 mg/mL) added per well. The stained cells were continuously incubated for 4 h at 37 °C. Next, MTT solution was removed and 100 µL of DMSO added per well. The resulting absorbance was measured at 570 nm using an automated microplate reader (ELx800, BioTek Instrument). The effect of liposomes on cell viability inside tumorspheres was calculated with Formula (2).

The IC_50_ was determined with Graphpad Prism 5.0 software, and the CI (combination index) value of L-asparaginase (ASNase)/cinchonain Ia was determined according to Chou (2006) [28]. The combination index was interpreted as additivity, antagonism, and synergism corresponding CI values of 0.9–1.1, >1.1, and <0.9, respectively. The *CI* was calculated using the formula:

(3) $$CI=\frac{Dx}{D\left(x\right)}+\frac{Dy}{D\left(y\right)}$$

where *Dx* and *Dy* are the concentration of compounds treated individually to access IC_50_; *D*(*x*) and *D*(*y*) are the concentration of compounds in combination to access IC_50_.

### 4.8. Antitumor Efficiency of CALs

The in vivo antitumor efficacy study was performed on tumorized BALB/c mice. Mice at 22–25 g were subcutaneously injected with 2 × 10^6^ LLC cells in 200 µL of culture medium in the right flank to induce tumors. On day 5 of LLC cell injection, mice were randomly divided into four groups (twelve mice per group). The control group was treated with unloaded-drug blank liposomes, and the three other groups were treated with CLs liposomes, ALs liposomes, and CALs liposomes. The dose of ASNase equivalence in Als- and CALs-treated groups was 3 IU/mouse. The drugs were administrated intravenously every 2 days, and in total seven injections were performed. Tumor volumes and body weight were measured every 7 days. The tumor volume was determined using a caliper in two dimensions, length (*L*) and width (*W*). The tumor volume (*V*) was measured using the formula [33]:

(4) $$V=\frac{1}{2}\times L\times W^{2}$$

The survival rate of tumorized mice in all experimental groups was recorded at the end of experiment and compared with the untreated control group.

### 4.9. Statistical Analysis

Statistical analyses of experimented results were performed using two-way analysis of variance (ANOVA). IC_50_ values were calculated with nonlinear regression using GraphPad PRISM 5.0 software (GraphPad Software, San Diego, CA, USA). In all comparisons, *p* < 0.05 was considered statistically significant.

## 5. Conclusions

This study is the first to manufacture cinchonain Ia/ASNase-loaded dual nanoliposomes and evaluate their bioactivity against NTERA-2 cancer stem cells. The CALs produced in this study exhibited strong synergistic anticancer potency, with CI values < 0.32 in 2D culture and 0.44 in 3D culture. The combination of these two drugs in nanolipsomal form exhibited greatly enhanced anticancer activity compared to either drug alone, both in vitro and in vivo. The antitumor effects of CALs led to approximately 62.49% tumor growth inhibition and 100% survival in tumorized mice by the end of the experiment.

## 6. Patents

The results from the work reported in this manuscript are in a patent named “L-asparaginaza and cinconain Ia nanoliposome complex with potential anti-tumor capacity”. The patent application is eligible and has the Decision number 2012w/QĐ-SHTT, issued by the Intellectual Property Office of Vietnam, dated 28 January 2022.

## Figures and Tables

**Table 1:** Antiproliferation activity of ASNase/cinchonain Ia combinations on some cancer cells at different sequences (*n* = 3 tests, average ± standard deviation).

Ratio of ASNase (IU):Cinchonain Ia (µg)	A549 Cells			NTERA-2 Cells		
	IC_50_ of ASNase (mIU/mL)	IC_50_ of Cinchonain Ia (µg/mL)	CI	IC_50_ of ASNase (IU/mL)	IC_50_ of Cinchonain Ia (µg/mL)	CI
1:0	30.3 ± 5.9	-	-	10.3 ± 0.6		-
0:1	-	100.73 ± 12.88	-	-	24.99 ± 4.25	-
1:12.5	21.5 ± 4.3 *	0.27 ± 0.05	0.71 ± 0.14	11.4 ± 0.5	0.15 ± 0.01	1.11 ± 0.05
1:25	22.3 ± 5.0 *	0.56 ± 0.12	0.74 ± 0.17	7.8 ± 1.6 *	0.19 ± 0.04	0.76 ± 0.16
1:50	21.3 ± 2.3 *	1.06 ±0.12	0.71 ± 0.077	5.8 ± 0.8 **	0.29 ± 0.04	0.57 ± 0.08
1:100	23.7 ± 2.2 *	2.37 ± 0.22	0.81 ± 0.08	6.1 ± 0.2 **	1.22 ± 0.05	0.64 ± 0.03
1:200	25.8 ± 1.9	5.15 ± 0.38	0.90 ± 0.07	12.3 ± 2.0	1.23 ± 0.20	1.24 ± 0.19

* *p* < 0.05, ** p < 0.01 compared with activity of ASNase.

**Table 2:** The characteristics of fabricated nanoliposomes.

Liposome Complex	Size (nm)	PDI	Zeta Potential (mV)	EE (%)
Blank	122.3	0.160	−25.8	-
CLs	127.9	0.190	−23.1	98.53
ALs	122.4	0.189	−30.5	93.75
CALs	118.7	0.120	−47.00	92.81 (ASNase) 99.97 (Cinchonain Ia)

**Table 3:** Average body weight of the experimented animals.

Groups		Body Weight (g)				
		Day 0	Day 7	Day 14	Day 21	Day 28
CLs	Mean	30.14	31.50	31.16	31.10	32.84
	SD	2.66	1.79	1.56	2.16	2.29
ALs	Mean	30.20	30.70	32.30	34.00	34.60
	SD	3.06	2.98	3.17	3.81	3.62
CALs	Mean	30.48	33.00	33.38	34.05	31.38
	SD	0.54	0.54	0.77	1.28	1.33
Control	Mean	29.98	32.44	32.98	33.42	32.81
	SD	1.57	1.15	1.29	1.61	1.29

**Table 4:** Efficacy of tumor growth inhibition of liposomes.

Time	Percentage of Tumor Growth Inhibition (%)		
	CLs	ALs	CALs
Day 0	-	-	-
Day 7	10.37	19.14	40.62
Day 14	32.93	48.34	43.67
Day 21	16.47	29.48	56.46 ^**, ##^
Day 28	16.24	27.21	62.49 ^***, ###^

** *p* < 0.01 and *** *p* < 0.001 compared with control; ^##^ *p* < 0.01 and ^###^ *p* < 0.001 compared with the respective uni-liposomal form.

## Data Availability

The data used to support the findings of this study are available from the corresponding author upon request.
